# Development of the endocrine pancreas and novel strategies for β-cell mass restoration and diabetes therapy

**DOI:** 10.1590/1414-431X20154363

**Published:** 2015-07-10

**Authors:** A.L. Márquez-Aguirre, A.A. Canales-Aguirre, E. Padilla-Camberos, H. Esquivel-Solis, N.E. Díaz-Martínez

**Affiliations:** Medical and Pharmaceutical Biotechnology, Center for Research and Assistance in Technology and Design of the State of Jalisco, A.C., Guadalajara, Jalisco, Mexico

**Keywords:** Endocrine pancreas, Diabetes mellitus, β-cell regeneration, β-cell replacement

## Abstract

Diabetes mellitus represents a serious public health problem owing to its global prevalence in the last decade. The causes of this metabolic disease include dysfunction and/or insufficient number of β cells. Existing diabetes mellitus treatments do not reverse or control the disease. Therefore, β-cell mass restoration might be a promising treatment. Several restoration approaches have been developed: inducing the proliferation of remaining insulin-producing cells, *de novo* islet formation from pancreatic progenitor cells (neogenesis), and converting non-β cells within the pancreas to β cells (transdifferentiation) are the most direct, simple, and least invasive ways to increase β-cell mass. However, their clinical significance is yet to be determined. Hypothetically, β cells or islet transplantation methods might be curative strategies for diabetes mellitus; however, the scarcity of donors limits the clinical application of these approaches. Thus, alternative cell sources for β-cell replacement could include embryonic stem cells, induced pluripotent stem cells, and mesenchymal stem cells. However, most differentiated cells obtained using these techniques are functionally immature and show poor glucose-stimulated insulin secretion compared with native β cells. Currently, their clinical use is still hampered by ethical issues and the risk of tumor development post transplantation. In this review, we briefly summarize the current knowledge of mouse pancreas organogenesis, morphogenesis, and maturation, including the molecular mechanisms involved. We then discuss two possible approaches of β-cell mass restoration for diabetes mellitus therapy: β-cell regeneration and β-cell replacement. We critically analyze each strategy with respect to the accessibility of the cells, potential risk to patients, and possible clinical outcomes.

## Introduction

The pancreas contains two principal components: the exocrine and the endocrine compartments. The exocrine pancreas consists of acinar and duct cells, the endocrine pancreas represents 2% of the pancreatic tissue and is organized into clusters of cells called islets of Langerhans. In mice, each islet is typically composed of five different cell subtypes: alpha, beta, delta, epsilon, and PP cells, which synthesize and secrete glucagon, insulin, somatostatin, ghrelin, and pancreatic polypeptide, respectively (1). When there are defects of insulin secretion, insulin actions, or both, the result is diabetes mellitus (DM).

DM is a metabolic disorder with multiple etiologies, characterized by chronic hyperglycemia complicated with disturbances in carbohydrate, fat, and protein metabolism. Two main forms of DM have been described: type 1 diabetes (T1D) and type 2 diabetes (T2D). T1D is an autoimmune disease characterized by the total loss of insulin-producing cells. T2D is the most prevalent form of DM (representing 90% of DM cases worldwide), and involves insulin resistance and/or a failure in insulin synthesis and secretion (2,3). In the last decade, there has been a significant increase in DM diagnoses, leading to the rise of DM as a major global public healthcare problem. There were an estimated 285 million people with DM in 2010, and the International Diabetes Federation predicts that 522 million will have DM by 2030 (4,5). To counter the effects of DM and the loss of functional insulin-producing cells, the administration of exogenous insulin is an important treatment for T2D and a life-saving therapy for patients with T1D (6). However, this treatment strategy is complicated because there is no physiological method to regulate glycemia. Mature β cells release insulin in proportion to blood glucose levels; however, exogenous insulin is not administered in relation to glucose concentration, leading to the deregulation of glycemia because of environmental variations such as exercise, diet, pregnancy, or age. Hypothetically, β-cell mass replacement is a curative strategy for DM because it restores the natural response to fluctuations in glucose levels, and insulin production and secretion. New treatments include whole pancreas or pancreatic islet transplantation, especially for the treatment of T1D patients. However, the scarcity of donors and the risks of surgery combined with treatment involving immunosuppressive drugs limit the clinical application of these approaches (7). Another strategy involves islet xenotransplantation (most frequently using porcine islets), which might be a promising approach for overcoming the disadvantages of allotransplants. However, the risk of immunologic rejection, acute inflammatory reactions, microangiopathy, systemic coagulopathy, and the potential transmission of endogenous porcine retroviruses, has limited the widespread application of these transplantation techniques (8).

Despite the recent improvements in DM care, there still is no effective cure for DM. Therefore, one of the most pressing objectives is to find new sources of β cells that can be used for replacement therapies. One such promising source is the generation of functional β cells from embryonic stem cells (ESCs), induced pluripotent stem cells (iPSCs), adult pancreatic cells, or cells isolated from adult tissues. This strategy might counter the total lack of naturally occurring β cells in T1D or the β-cell mass deficiency in T2D. For β-cell replacement therapy to be successful, an understanding of β-cell development during embryogenesis and postnatal maturation is required.

In this review, we summarize the current knowledge of mouse pancreas organogenesis, morphogenesis, and maturation, and we explored the molecular mechanisms involved at each step. We then discuss two potential approaches for β-cell mass restoration in DM therapy: 1) β-cell regeneration, including proliferation, neogenesis, and transdifferentiation, and 2) β-cell replacement, including transplantation of insulin-producing cells that are differentiated (or transdifferentiated) from ESCs, iPSCs, or non-pancreatic adult cells into insulin-producing cells. We critically analyze each strategy with respect to the accessibility of the cells, potential risk to patients, and possible clinical outcomes and success.

## Pancreatic organogenesis and morphogenesis

### Embryonic pancreas development

Pancreatic development is a complex and highly regulated process that controls the specification and differentiation of progenitor cells, and is guided by multiple signaling pathways and transcription factor cascades (8). The first step of pancreatic development is primitive endoderm (PrE) specification from pluripotent stem cells isolated from the mural surface of the inner cell mass of blastocysts (3-5 days post-fertilization in mice). The PrE consists of extraembryonic endoderm precursor cells characterized by *Sox7* expression that subsequently differentiate into visceral endoderm and parietal endoderm (9). Shortly after PrE specification, gastrulation occurs to generate the three germ layers: ectoderm, mesoderm, and endoderm. Definitive endoderm (DE) cells (formed between embryonic day (E) 6.5 and E7.5 in mice) co-express the transcription factors *Foxa1* and *Foxa2.* DE cells then form gastrointestinal organs such as the liver, lungs, thymus, respiratory tract, digestive tract, and pancreas. However, when the endoderm initially differentiates, it is not committed to specific cell or tissue lineages. Therefore, the second important specification step towards pancreatic fate occurs when DE cells form the posterior gut endoderm, which develops into the midgut and hindgut, and subsequently, the intestine. The transcription factors *Pdx1* and *Ptf1α* are expressed at the foregut-midgut junction. *Pdx1*-positive cells were shown to contribute to the formation of the endocrine and exocrine compartments. Similarly, all *Ptf1α*-positive cells generate pancreatic derivatives ([Bibr B10] [Bibr B11] [Bibr B12] 1013). Posterior and anterior foregut endoderm develops into ventral and dorsal pancreatic buds around E9 and E9.5, respectively. The ventral bud is surrounded by cardiac mesenchyme, while the dorsal bud is in contact with the notochord. These interactions with mesoderm-derived neighboring tissues regulate pancreas organogenesis and drive the subsequent specification steps (14). Morphogens regulating this process include fibroblast growth factor and activin produced by the notochord that signals to the dorsal pancreatic bud to repress Sonic Hedgehog and a Hedgehog signaling pathway ligand. Fibroblast growth factor and bone morphogenic proteins signal from the cardiac mesoderm to the ventral pancreatic bud (15,16). It should be noted that the distinct origins of the pancreatic buds have an impact on pancreatic organogenesis later in development.

**Figure 1:**
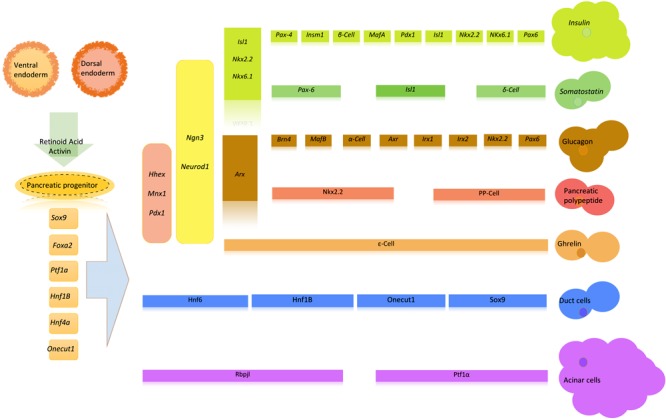
Schematic of pancreatic progenitors toward differentiated lineages. Upon activation of PDx1, the pancreatic fate is induced from endoderm progenitors. Pancreatic progenitors give rise to acini, ductal, and endocrine progenitors. Endocrine progenitors then differentiate into specific hormone secreting cells: α, β, δ, PP, and ε cells. Key transcription factors involved in each differentiation step and the time they are expressed are indicated.

Around the E9.5 stage, the pancreatic buds are formed from multi-potent progenitors that contribute to all cell types in the pancreas. These epithelial buds invade the surrounding mesenchyme by subsequent waves of branching morphogenesis called the primary and secondary transition. The primary transition (occurring between E9.5-E12.5) is a period of active pancreatic progenitor proliferation, followed by expansion of the epithelial network to achieve organ determination. Concomitant with this stage, the first endocrine cells are detected (17). At E11.5, gut rotation brings the two buds into proximity, allowing their subsequent fusion around E17-18. At E12.5, invaginations of the pancreatic epithelium appear in the surrounding mesenchyme, initiating epithelial compartmentalization into the tip (primarily of acinar cell origin) and trunk (of endocrine and duct cell origin, and determined by *Pdx1*, *Ptf1a*, *Cpa1*, and *C-myc* expression) domains. In the secondary transition (E13.5-E16.5), the morphogenetic transformation of pancreatic epithelium occurs. This period is characterized by the specification of multi-potent progenitors toward differentiated lineages, a process achieved by the initiation and maintenance of specific gene expression profiles controlled by distinct spatial and temporal combinations of transcription factors (Figure 1). Importantly, endocrine cell specification and subsequent differentiation occurs via the inhibition of Notch signaling, leading to the expression of the pro-endocrine gene Neurogenin 3 (*Ngn3*) in some pancreatic epithelial cells. At E13, a wave of basic helix-loop-helix transcription factor, *Ngn3*, expression in trunk epithelium leads to the differentiation of endocrine cell expansion between E13 and E15 by triggering the expression of several transcription factors including *Nkx2.2*, *Neurod1*, *Nkx6.1*, *Pax4*, *Pax6*, and *Isl1,* which control endocrine cell differentiation. Over the next several days (E14-E18), endocrine cells begin pancreatic islet morphogenesis by coalescing into small aggregates of cells ([Bibr B18] [Bibr B19] 1820). However, the final adult architecture of Langerhans islets is not fully formed until after birth.

### Postnatal maturation of pancreatic islets

Emerging strategies for the treatment of DM, including β-cell regeneration and replacement, rely on knowledge of β-cell development and maturation. This is a challenging and unresolved issue, and is now recognized as an important topic. Early postnatal pancreatic development is important for adults to achieve effective glycemic control. Defects in β-cell maturation are thought to promote the development of metabolic diseases such as DM. Similarly, a failure in the expansion of β-cell mass determines susceptibility to DM (21).

Pancreatic islet cell differentiation occurs during the various embryonic stages; however, β-cell maturation only occurs after weaning. Two crucial maturation events are required to have functional β cells: *1*) glucose sensing machinery is enhanced when insulin production per cell changes, leading to increases in insulin-containing dense core secretory granules. This results in the maturation of stimulus-secretion coupling (22,23). *2*) an appropriate β-cell mass is established and expands in proportion to an individual’s body weight and pancreatic islet remodeling, leading to morphological maturation (24,25).

**Figure 2:**
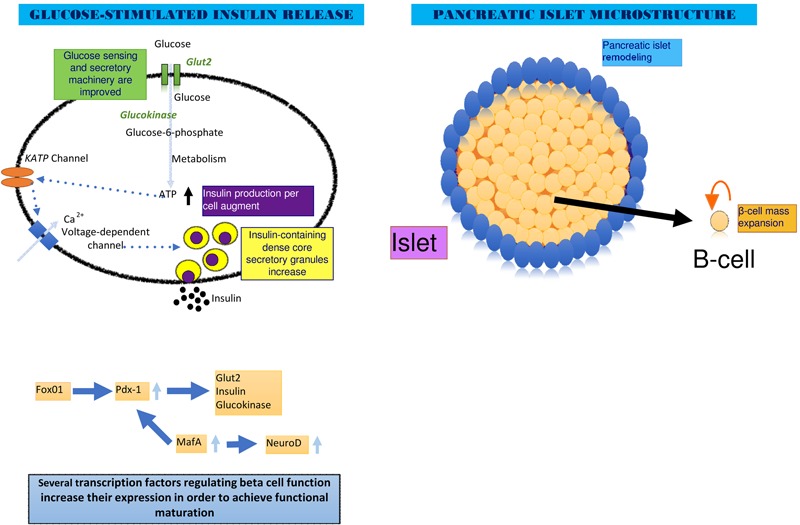
Functional and morphological postnatal pancreatic maturation. After weaning, normal β-cell development culminates in two crucial maturation events: the *left panel* shows glucose sensing machinery is enhanced when insulin production per cell changes, leading to increases in insulin-containing dense core secretory granules. This results in the maturation of stimulus-secretion coupling. The *right panel* illustrates the establishment of appropriate β-cell mass in proportion to an individual’s body weight and pancreatic islet remodeling, leading to morphological maturation. Overexpression of key genes involved in the maturation process is indicated.

Several genes are involved in postnatal β-cell maturation. To achieve maturation and β-cell stimulus-secretion coupling during the first postnatal weeks, β cells increase their expression of genes encoding hallmark factors including: preproinsulin and insulin (genes involved in the maintenance of islet cell identity); glucose transporter 2 (*Glut2*) and glucokinase (*Gk*) (glucose sensing machinery genes); *Pdx1*, *MafA*, and *NeuroD* (transcription factors important in the development and function of mature β cells); chromogranins (Chg)A and ChgB, and islet amyloid polypeptide *(IAPP*) (genes involved in the formation of secretory granules); *SUR1* (one of four regulatory sulfonylurea receptors in K(ATP) channels), *Kir6.1* (one of four K(ATP) ion channels) and calcium channel type 1D (genes participating in glucose-induced insulin secretion); and pyruvate carboxylase, mitochondrial glycerol-3-phosphate dehydrogenase, mitochondrial malate dehydrogenase 1 and 2 and aspartate aminotransferase (important genes in the maintenance of the specialized β-cell metabolic phenotype related to glucose-induced insulin secretion) (23-26). To achieve morphological maturation, the expression of several genes involved in β-cell proliferation including cyclin dependent kinase 4, *CyclinD2*, and the transcription factor *FoxM1* (27,28) are enhanced (Figure 2). These protein levels are at their highest level in neonatal mice but decline in adults.

During the maturation process, the expression of several genes in β cells increases or is repressed, ensuring their proper function. One of the genes most highly repressed during the first postnatal weeks is *Mct1*, encoding the monocarboxylate transporter MCT1, which mediates the transport of pyruvate and lactate across cell membranes. This repression prevents the potential for hypoglycemia after physical exercise caused by inappropriate insulin release. Lactate dehydrogenase, which catalyzes the conversion of lactate to pyruvate, is also repressed in β cells as an additional safeguard to ensure insulin is released exclusively in response to glucose (29,30). Thus, an immature glucose metabolism system might account for the lack of glucose responsiveness in neonatal β cells, but the mechanism of how glucose-stimulated insulin secretion is acquired during the postnatal period is still largely unknown.

Different studies have demonstrated that transient calcium induced by *Gk* activation enhanced the production of insulin, insulin secretion, and β-cell proliferation (31). As previously stated, *Gk* mRNA activity increases during the postnatal period; thus, *Gk* and calcium signaling may be physiological regulators of pathways that govern β-cell functional and morphological maturation. Goodyer et al. (32) demonstrated that calcineurin/nuclear factor of activated T cells (NFATc) signaling regulated neonatal pancreatic development in mouse and human islets through the regulation of genes coordinating β-cell maturation (*Glut2*, *Pdx1* and *Gk*), dense core secretory granule formation (*ChgA/B*, *IAPP*, and *IA2*), and β-cell proliferation (*Cd2* and *FoxM1*). Calcineurin is a Ca^2+^-activated serine/threonine phosphatase required for the activation of the NFATc family of transcription factors. Following a sustained increase in intracellular Ca^2+^, calcineurin activation leads to the dephosphorylation of NFATc proteins, which allows the nuclear translocation of NFATc and the regulation of gene transcription (33).

## Novel treatments for diabetes mellitus

### β-cell regeneration

Understanding the mechanisms underlying pancreas organogenesis and maturation identifies new opportunities for the development of novel approaches for DM treatment. Of considerable interest are methods designed to restore β-cell mass *in vivo*, avoiding the complications of tissue matching and surgical procedures. To date, a number of different models and mechanisms to induce endocrine cell regeneration have been proposed, such as proliferation of pre-existing adult β cells, neogenesis (β-cell differentiation from progenitors within the pancreas), and transdifferentiation (conversion from non-β cells within the pancreas to β cells).

#### Proliferation

Proliferation is the expansion of pre-existing adult β cells through cell division. In the rodent pancreas, 2%-3% of total β cells replicate every 24 hours, accounting for their slow turnover during adult life. However, the regeneration of pancreatic β cells *in vivo* indicated the high proliferation capacity of postnatal β cells in situations of increased metabolic demand (34). For instance, during pregnancy, β-cell mass can expand due to the action of circulating maternal hormones (prolactin and placental lactogen). In addition, β-cell mass increases with age, and the replication rate changes significantly during life (from 20% per day in mouse pups to over 10% at weaning, and then declining to 2%-5% in young adults and 0.07% in one year old mice). Similarly, adult β cells can expand under conditions of obesity (35). In animal models, many studies on the regeneration of pancreatic β cells have been performed. Studies using different models of pancreatic injury, including pancreatectomy, diphtheria-toxin-induced β-cell ablation, and pancreatic duct ligation, revealed that regeneration might occur in the adult pancreas. Genetic and DNA analog-based lineage tracing experiments have provided strong evidence that the division of pre-existing β cells is the major mechanism by which regeneration after trauma is achieved (36,37). Growth factors are used to stimulate pancreatic β-cell proliferation *in vivo*. Molecules such as hepatocyte growth factor, epidermal growth factor (EGF), betacellulin, and connective tissue growth factor, which can stimulate β-cell proliferation and insulin production, have been investigated as potential therapies for DM. It is important to note that the therapeutic potential of these approaches in humans has not been explored in sufficient detail to make firm conclusions (38,39). Similar to the use of growth factors, several hormones are implicated in regulating β-cell proliferation. The transgenic expression of parathyroid hormone-related protein in β cells increased their mass as well as insulin secretion. Furthermore, several studies in various mouse models of DM and obesity demonstrated that GLP-1 and its analogs, such as exendin-4, could induce β-cell regeneration and improve glucose tolerance. Another gastric hormone, gastrin, has also been implicated in regulating β-cell proliferation. When used in conjunction with other factors such as EGF and GLP-1, it increased β-cell mass ([Bibr B40] [Bibr B41] [Bibr B42] 4043). In addition to growth factors and hormones, small-molecule inducers of pancreatic β-cell expansion have been investigated for their potential to stimulate proliferation. Recently, Wang et al. (44) reported a high-throughput chemical library screening for inducers of β-cell proliferation (44). They used growth-arrested, reversibly immortalized mouse β cells, and found various molecules that promoted β-cell proliferation, including novel Wnt signaling and L-type calcium channel (LTCC) agonists. The LTCC agonist induces replication by activating Ras signaling, and the co-treatment of β cells with the LTCC agonist and exendin-4 showed an extended effect on β-cell proliferation. However, the stimulation of endogenous β-cell proliferation by small molecules or biological signals is not yet ready for clinical application.

#### Neogenesis

As mentioned previously, it was reported that proliferation of β cells is the principal mechanism by which regeneration after pancreatic injury is achieved; however, it was argued that neogenesis (β-cell differentiation from progenitors within the pancreas) also contributed to increased β-cell mass during normal growth and after trauma ([Bibr B45] [Bibr B46] [Bibr B47] 4548). Neogenesis has recently been a source of intense debate, and cell-tracing studies, together with histological analysis, have reported contradictory results. Dor et al. (38) selectively labeled β cells by Cre-loxP-based conditional recombination in the adult pancreas and chased the fate of pre-existing β cells. They concluded that new cells were generated primarily from pre-existing β cells, casting doubt on the significance of adult progenitor cells in pancreatic regeneration. Furthermore, Teta et al. (49) used a lineage-tracing technique to show that, unlike gastrointestinal and skin epithelia, specialized progenitors do not contribute to adult β-cell mass, even during acute β-cell regeneration. Instead, mature β cells displayed equal proliferation rates and expanded from within a vast and uniform pool of adult β cells. Kopp et al. (50) described the derivation of non-β endocrine cells from the ducts in early postnatal life, but no endocrine or acinar cell neogenesis occurred in adult mice either physiologically or after pancreatic duct ligation.

Conversely, there are several reports describing pancreatic adult stem/progenitor cells *in vivo*. When both acinar and islet cells were killed *en masse* by diphtheria toxin expressed under the *Pdx1* promoter, duct cells gave rise to acinar and endocrine cells, recovering 60% of the β-cell mass; but, when only acinar cells were eliminated by elastase-driven toxin, duct cells only gave rise to acinar cells. Furthermore, neogenesis from the ducts occurred in a pancreatic duct ligation model in mice and in a partial pancreatectomy model in rats. Xu et al. (51) demonstrated that the differentiation of adult progenitors is *Ngn3*-dependent and gives rise to all islet cell types, both *in situ* and *in vitro*. Moreover, several reports showed that islet neogenesis associated protein-pentadecapeptide (INGAPPP) stimulated neogenesis and reversed DM in streptozotocin treated mice (52,53). In the human pancreas, indirect evidence of neogenesis has been provided by the presence of cells containing insulin within the ducts. In addition, the role of neogenesis during pregnancy was demonstrated: a recent autopsy study on the pancreas showed an increased relative volume of β cells, an augmented proportion of small islets, and increased numbers of insulin-positive cells within the ducts. However, no changes in β-cell proliferation, cell size, or apoptosis frequency were observed. Similarly, Inada et al. (54) reported that after birth, a subset of adult β cells was generated from pancreatic duct cells ([Bibr B54] [Bibr B55] [Bibr B56] [Bibr B57] 5458). An increased understanding of the neogenesis process was also obtained using genetic manipulation techniques. The overexpression of transforming growth factor-α (TGF-α) induced the expansion of *Pdx1*-expressing ductal cells, leading to an increase of focal areas of islet neogenesis (59).

These results clearly highlight the technical limitations of the current lineage tracing approaches. We therefore conclude that neogenesis is determined by the type and extent of pancreatic injury. Currently, the general concept is that, after birth, neogenesis occurs mostly during the neonatal period and can also be stimulated following pancreatic injury.

#### Transdifferentiation

We define transdifferentiation as the conversion of a differentiated cell from one developmental lineage into a differentiated cell of another lineage. There have been reports of α- to β-cell conversion in response to severe pancreatic injury. Thorel et al. (60) selectively expressed the diphtheria toxin receptor downstream of the rat insulin promoter. Following 99% β-cell ablation, α cells were found to pass through a bi-hormonal state (cells expressing both insulin and glucagon) prior to acquiring a single-hormone insulin-positive cell identity. This suggested that regeneration occurred from a non-β-cell source, demonstrating the contribution of α cells to β-cell mass restoration after injury. Chung et al. (61) also reported α to β-cell conversion in response to pancreatic injury. They used pancreatic duct ligation in conjunction with alloxan treatment to ablate β cells, and observed a rapid β-cell differentiation from α cells, resulting in the formation of islets within two weeks. Furthermore, the genetic reprogramming of α cells into cells with a β-cell phenotype has been demonstrated (62). α cell-specific *Men1* knock-out mice showed a conversion of α cells into β cells. The transgenic expression of *Pdx1* in *Ngn3* positive cells, and the expression of *Pax4* in α cells, also triggered the transdifferentiation of α cells into β cells and reversed the effects of chemically induced DM (62). Other attempts using small molecule drugs (such as GW8510) to induce transdifferentiation successfully predisposed α cells to adopt various features of β cells; however, a detailed mechanism remains unknown (63).

In addition to α cells as an effective source of β cells, acinar cells might also be converted into β-like cells. Acinar to endocrine conversion was demonstrated using *in vitro* cultured primary acinar cells treated with growth factors such as EGF and leukemia inhibitory factor (64). Furthermore, the enforced simultaneous expression of three key developmental transcription factors, *Pdx1*, *Ngn3*, and *MafA*, induced acinar to β-cell conversion and rescued hyperglycemia in streptozotocin-induced diabetic animals (65). However, Pan et al. (66) recently demonstrated that acinar cells, without exogenously introduced factors, could be converted into mature β cells after injury. They used a knock-in, tamoxifen inducible, lineage-tracing method using multipotent progenitor cell-instructive gene *Ptf1α*, to define the role of acinar cells in the restoration of β-cell mass after pancreatic duct ligation and streptozotocin-induced elimination of pre-existing insulin-positive cells. In this model, pancreatic injury caused the facultative reactivation of multipotent transcription factors such as *Sox9* and *Hnf1α* in *Ptf1α*-positive acini, which underwent reprogramming to produce duct cells and longer-term reprogramming to produce endocrine cells, including insulin-positive cells. These insulin-positive cells were considered mature based on their expression of *Pdx1*, *Nkx6.1*, and *MafA*. However, the clinical significance of the transdifferentiation process must still be demonstrated.

### β-cell replacement therapy

Because human islet transplantation is limited by the scarcity of donors, efforts have currently concentrated on exploring new potential sources of β cells. The use of pluripotent ESCs, non-pancreatic adult cells, and iPSCs for the *in vitro* differentiation and expansion of insulin-producing cells represents an attractive strategy for obtaining a large number of β cells for transplantation. For this reason, generating pancreatic β cells in culture using these sources is a major research topic focusing on the concepts of developmental biology.

### Differentiation of insulin-producing cells from embryonic stem cells

The first report of stem cell isolation was from mice, and stem cell research has been a central focus of many developmental biology laboratories over the last 30 years; however, the use of human stem cells is more recent, especially in the field of pancreatic stem cell derivation. The use of stem cells has many advantages over other cell sources because these unique cells proliferate at a high rate, they are readily available, and they retain the potential to differentiate into derivatives of all three embryonic layers: endoderm, ectoderm, and mesoderm. Indeed, ESCs can initiate a differentiation process to generate β cells when they are cultured according to very precise protocols of pancreatic specification induction, representing a new research avenue in β-cell replacement therapy (67).

In the last decade, efforts have been directed at developing efficient protocols for the differentiation of ESCs to mature insulin-producing cells. The expansion of pancreatic β cells from ESCs represents an attractive strategy, and has been successful in obtaining a large number of β cells with the ability to store and secrete insulin in a regulated manner in response to glucose demand *in vitro* (68). Lumelsky et al. (69) first reported the generation of β-like cells from mouse ESCs in 2001. Their cell differentiation protocol was based on the production of a highly enriched population of nestin-positive cells from embryoid bodies (EBs). The critical step in this approach consisted of plating the EBs into serum-free medium, in which many other cell types die, increasing the proportion of nestin-positive cells. Finally, nicotinamide supplementation and B27 culture media was necessary to improve the yield of pancreatic endocrine cells and the expression of three other pancreatic endocrine hormones, glucagon, somatostatin, and pancreatic polypeptide. In contrast, Assady et al. (70) showed that spontaneous differentiation from human ESCs *in vitro* under two conditions, adherent culture and EBs, resulted in the generation of cells with similar insulin-producing characteristics. These cells synthesized and secreted insulin and expressed essential genes for β-cell differentiation and function, such as *Glut2*, *Gk*, and *Pdx1*. However, because these insulin positive cells showed poor insulin secretory responses, they could not be categorized as β cells. Subsequently, Maria-Engler et al. (71) analyzed the expression of β-cell markers during short- and long-term islet cell cultures derived from different human islet preparations. Using confocal microscopy and RT-PCR they demonstrated the rare co-localization of nestin and insulin, both in freshly isolated islets and in long-term cultures enriched with nestin-positive cells obtained from cadaveric donors. This suggested that these cells could be undergoing the early stages of differentiation to a β-cell phenotype and that they might proliferate at high rates, as determined by the proportion of BrdU-incorporation. Low levels of insulin, glucagon, and somatostatin mRNA were detected after prolonged subculture in low serum medium and Matrigel without the addition of differentiation-inducing factors. This indicated that nestin-positive cells might be considered as a potential source of precursor cells to generate fully differentiated and functional β cells, despite the existence of different nestin-positive progenitor cells other than those pancreatic epithelial cell progenitors previously described. Years later in 2006, D’Amour et al. (72) developed a new method to generate insulin-secreting cells. In this method, the differentiation processes mimicked pancreatic organogenesis by directing ESCs through different stages resembling definitive pancreatic endoderm formation (expressing the markers *Sox2*, *Sox17*, and *FoxA2*) using endocrine precursors rather than the visceral endoderm. Accordingly, most of the current protocols for ESC differentiation into β cells involve many of the developmentally active signaling pathways, such as Wnt and TGF-β, and growth factors, such as activin, fibroblast growth factor 10, and retinoic acid, leading to the expression of endodermal markers and subsequent *Pdx1* expression. It is important to note that in recent and improved studies, other markers were found to identify definitive endoderm, including Sox17, Brachyury protein, CXC-chemokine receptor type 4, and Cerberus (73). In all of the assays previously described, the source of cells was from the inner cell mass of mouse or human blastocysts.

To develop more efficient differentiation protocols, many groups have attempted to identify small molecules that might control the process of differentiation by the modulation of gene expression or metabolism (74). Other approaches consist of replicating the formation of the dorsal pancreatic anlage, which depends on simultaneous retinoic acid signaling and inhibition of Hedgehog signaling (17).

It is important to note that the clinical use of ESCs is still hampered by ethical issues as well as the risk of *in vivo* teratoma formation associated with the transplantation of cells with undifferentiated phenotypes (75). Efforts are now being concentrated on the specific selection of differentiated cells only. Recent studies demonstrated that sorting ESC-derived endodermal cells using cell surface markers was possible and that no detectable teratoma formation was observed at 160 days post-transplantation (76,77). However, whether the differentiated products might revert to a less differentiated and potentially dangerous state is unknown.

The most recent development in this line of research was reported by Pagliuca et al. (78) where they described the large-scale *in vitro* production of functional human β cells (glucose-responsive, mono-hormonal, insulin-producing cells that co-express key β-cell markers and have normal β-cell ultrastructure) from human pluripotent stem cells using sequential modulation of multiple signaling pathways in a three-dimensional cell culture system without transgenes or genetic modification. Furthermore, these cells secreted human insulin into the serum of mice shortly after transplantation in a glucose-regulated manner, ameliorating hyperglycemia in a DM mouse model. Importantly, this is the only protocol that has generated full, mature β cells in significant amounts, demonstrating the potential utility of these cells for *in vivo* transplantation therapy for DM treatment (78).

### Differentiation of insulin-producing cells from induced pluripotent stem cells

The generation of iPSCs has emerged as a unique cellular system, and is increasingly becoming an interesting model system in developmental biology and regenerative medicine. Currently, there are various methods for reprogramming a somatic cell to become a pluripotent cell by the ectopic overexpression of transcription factors. iPSCs are reprogrammable, and differentiate into several cell types both *in vitro* and *in vivo.* They also allow for the potential generation of autologous cells that may be useful for clinical therapy, as they do not exhibit immunorejection when grafted back to the donor (79). Moreover, because of their high proliferative capacity, they can be used to produce a large number of differentiated cells. These features make iPSCs an excellent alternative source of β cells for replacement therapy. iPSCs are also an important model for the investigation of the etiology of metabolic diseases.

For instance, the differentiation of human skin fibroblast cells by the retroviral expression of *Oct4*, *Sox2*, *c-Myc*, and *Klf4* using a serum-free differentiation procedure, is sufficient to generate insulin-producing islet-like clusters. These iPSCs express *Pdx1*, *Foxa2*, and *Sox17* and release C-peptide upon glucose stimulation, showing that the generation of patient-specific iPSCs with potential for DM treatment is possible (80). In another assay, Maehr et al. reported the isolation of iPSCs obtained from patients with T1D (81). Their reprograming process used three factors, *Oct4*, *Sox2*, and *Klf4*; however, the reported differentiation efficiency was low. To improve differentiation techniques, Zhang et al. (82) developed a new protocol that increased the efficiency of human ESC differentiation by using EGF in a population of *Pdx1* positive cells, which subsequently differentiated into a final cellular stage expressing *Pdx1*, *MafA*, *Glut2*, and *insulin*. Furthermore, Thatava et al. (83) stimulated human iPSC differentiation in feeder free conditions using the pancreatic endoderm inducer, indolactam V, in combination with GLP-1. Conversely, Alipio et al. (84) showed the rescue of two mouse models of T1D and T2D via mouse iPSC transplantation. In this study, the reprogramming process was activated using *Oct4*, *Sox2*, *Klf4*, and *c-Myc*, and iPSCs were subjected to a three-stage differentiation protocol.

Safety issues were recently been raised because coding mutations and epigenetic anomalies have been observed after reprogramming (85,86). Undefined limitations also exist because it is not yet possible to induce iPSC differentiation without generating large numbers of undifferentiated cells. To develop safer, non-integrative techniques, Anokye-Danso et al. (87) reported the successful reprogramming of mouse and human somatic cells using miR302/367 microRNAs. More efficient and faster reprogramming was obtained using non-integrative episomal vectors on bone marrow and cord blood cells (88). Further work is needed to confirm the reliability and safety of these protocols prior to clinical application.

### Differentiation of insulin-producing cells from non-pancreatic adult cells

Because islet donors are scarce, human β cells for therapeutic use might be obtained by expanding non-pancreatic tissues *in vitro*. Mesenchymal stem cells (MSCs) are pluripotent stromal cells that proliferate and differentiate into a variety of cell types, including endocrine cells of the pancreas. For example, human adipose-derived tissue obtained from liposuction aspirates were induced to differentiate into insulin-secreting cells *in vitro* using a combination of three factors: β-mercaptoethanol, nicotinamide, and exendin-4. These β cells possessed typical morphology and expressed several transcription factors and other genes involved in endocrine pancreas development and function, such as *Pdx1*, *Pax4*, *Ng3* and *Glut-2* (89). Furthermore, Gabr et al. (90) obtained bone marrow cells from adult T2D volunteers and non-diabetic donors to perform a three-staged differentiation procedure without genetic manipulation. They demonstrated the formation of insulin producing cells and reported control over diabetic status after transplantation of β cells into nude diabetic mice. However, conclusive *in vitro* studies are necessary to understand the potential of mesenchymal stem cells for therapy.

## Conclusions

**Figure 3:**
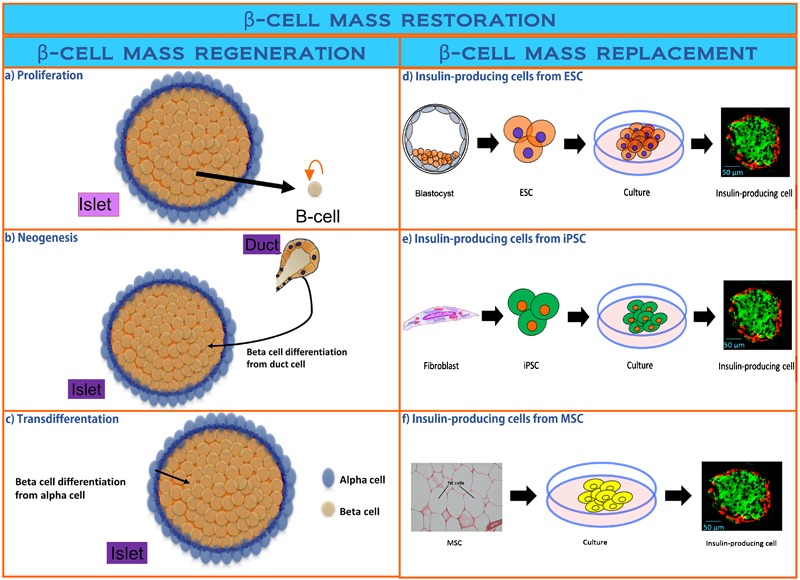
Different strategies for β-cell mass restoration. Novel strategies for β-cell mass restoration in diabetes therapy can be divided into the following groups: 1) β-cell regeneration, which includes proliferation (*a*), neogenesis (*b*), and transdifferentiation (*c*), and 2) β-cell replacement, which involves the transplantation of insulin-producing cells differentiated from embryonic stem cells (ESC) (*d*), induced pluripotent stem cells (iPSCs) (*e*), and mesenchymal stem cells (MSC) (*f*).

Novel strategies for β-cell mass restoration in DM therapy can be divided into the following groups: 1) β-cell regeneration and 2) β-cell replacement, which involves the transplantation of insulin-producing cells differentiated from embryonic stem cells, iPSCs, and non-pancreatic adult cells (Figure 3). β-cell regeneration should restore β-cell mass *in vivo*, avoiding complications involved with tissue matching and surgical procedures. Thus, different models and mechanisms of endocrine cell regeneration have been proposed: β-cell proliferation, neogenesis, and transdifferentiation. β-cell replacement therapy is also a promising field of research that is currently evaluating new sources of cells, such as ESCs, iPSCs, MSCs, and cord-blood-derived stem cells, for clinical use.

Recent findings have opened new research avenues in the field of DM therapy. There is reason to be optimistic that an efficient β-cell mass restoration protocol will be available soon. Stimulating the *in situ* regeneration of β cells might be a less invasive procedure with high clinical value. However, therapy with cells derived from stem cells has gained attention after high levels of cellular differentiation were obtained with ESCs and iPSCs. However, safety is a critical issue with these cell types and might delay their clinical application. New insights into progenitor or somatic cell differentiation have opened the door for future investigations, but *in vitro* and *in vivo* evaluation is necessary to understand their potential for therapy.
